# Constructing module maps for integrated analysis of heterogeneous biological networks

**DOI:** 10.1093/nar/gku102

**Published:** 2014-01-31

**Authors:** David Amar, Ron Shamir

**Affiliations:** Blavatnik School of Computer Science, Tel Aviv University, Tel Aviv 69978, Israel

## Abstract

Improved methods for integrated analysis of heterogeneous large-scale omic data are direly needed. Here, we take a network-based approach to this challenge. Given two networks, representing different types of gene interactions, we construct a map of linked modules, where modules are genes strongly connected in the first network and links represent strong inter-module connections in the second. We develop novel algorithms that considerably outperform prior art on simulated and real data from three distinct domains. First, by analyzing protein–protein interactions and negative genetic interactions in yeast, we discover epistatic relations among protein complexes. Second, we analyze protein–protein interactions and DNA damage-specific positive genetic interactions in yeast and reveal functional rewiring among protein complexes, suggesting novel mechanisms of DNA damage response. Finally, using transcriptomes of non–small-cell lung cancer patients, we analyze networks of global co-expression and disease-dependent differential co-expression and identify a sharp drop in correlation between two modules of immune activation processes, with possible microRNA control. Our study demonstrates that module maps are a powerful tool for deeper analysis of heterogeneous high-throughput omic data.

## INTRODUCTION

Biological networks provide a comprehensive overview of biological systems. They enable better understanding of the system and can shed light on the function of genes and other molecular compounds. Among other applications, they have been used for discovery and prediction of gene interactions, gene functions and disease–gene associations (1–9).

In these networks, the nodes represent molecular entities and the edges represent interdependencies. For example, in protein–protein interaction (PPI) networks, nodes represent proteins and edges represent physical interactions. In genetic interaction (GI) networks, nodes represent genes and edges represent the organism fitness for double-knockout perturbations, yielding two major types of edges: alleviating GIs and aggravating GIs. In alleviating GIs, also called positive GIs, the organism fitness after the double-knockout perturbation is better than expected based on the single-knockout results. In aggravating or negative GIs, the fitness is worse than expected. In gene co-expression networks, nodes represent genes and edges score the correlation in expression between the two genes (10,11). In gene differential correlation (DC) networks, edges score the change in gene pairwise correlation between one set of samples to another (e.g. cases and controls) (12–14). With the growing use and number of types of biological networks, computational methods that exploit these rich data are of great importance.

Computational methods that make use of several networks are often better than methods that analyze only a single network (4,7,8,15–19). For example, combined analysis of PPI networks and gene co-expression networks was used to detect gene sets that are co-expressed and are connected in the PPI network. Such analysis outperformed standard clustering algorithms and was successfully used for gene function prediction (5,8,16,19). Alleviating and aggravating GI data were used to find epistasis among and within gene sets. Under the premise that negative GIs tend to occur between compensatory pathways and positive GIs occur within pathways (or complexes), analysis of GIs was used to suggest a map of epistatic relations among functional gene modules (15,17,20–23). A marked improvement was reported after adding a connectivity constraint in a PPI network of the modules (15,17). The ability to construct a summary map of several networks allows identifying associations among discovered modules, thus improving the interpretability of the results compared with standard clustering of a single network.

**Figure 1.**
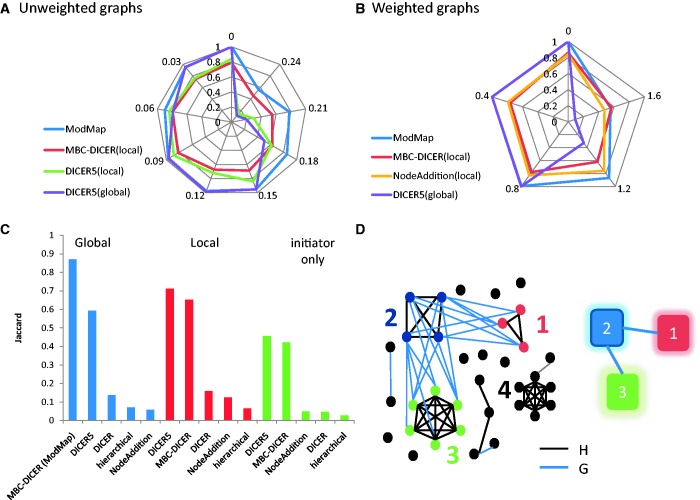
Module map: example and simulation results. (**A** and **B**) Performance of module map algorithms on 500-node graphs. (A) Unweighted graphs. (B) Weighted graphs. Each simulated pair of graphs contained an embedded module map of six modules in a tree structure. In addition, two random cliques and two bicliques were embedded in the graphs as decoys. Module, clique and biclique size was chosen uniformly at random between 10 and 20. In the unweighted model (A) each edge was replaced by a non-edge with probability *P* and vice versa. In the weighted model (B) edge weights are sampled from the normal distribution N(1,σ), and non-edge weights are sampled from the normal distribution N(−1, σ). Results are averages of 10 simulations for each data point. The four top performing algorithms for each simulation are presented using radar plots. MBC-DICER with global improvement is denoted as ModMap. The Jaccard coefficient between the modules produced by each algorithm and the true modules is shown as the distance from the center. Consecutive spokes from the top anticlockwise show increasing values of *P* in A and of σ in B. (**C**) Comparison of module map algorithms on unweighted graphs with 1000 nodes, containing a map of 10 modules and five decoys and *P* = 0.15. (**D**) A toy example of the module map problem; left: the two networks. Nodes are genes, H edges are black and G edges are blue; right: the module map. Nodes are modules and edges are links. Colors and numbers are the same on the left and right. The map contains three modules: module 2 is linked to modules 1 and 3, whereas module 1 and 3 are not linked. Black nodes are not part of the module map. The graph H (black edges) contains a clique that is not linked in G to another module and thus is not a part of the map. The example also demonstrates the difference between the local and global approaches. The local approach identifies modules 1 and 2 as linked, whereas the global approach also identifies module 3 as linked to module 2. See text.

Building on prior studies of specific pairs of networks, we introduce and study the fundamental problem of constructing a summary map of two biological networks H and G, where the nodes of both are the same genes or proteins, and the edges in each represent a distinct type of relations (see Figure 1D). The map nodes are gene sets that are strongly connected in H, and pairs of sets are connected by links. A link represents strong connection between two gene sets in G. The goal is to find gene modules in H simultaneously with finding module-to-module interactions according to G, by optimizing a specific objective function. We call this computational problem the ‘module map problem’*.*

Most algorithms for the module map problem to date were used to find a summary map of epistatic interactions among pathways (15,17,20–23). Kelley and Ideker (15) proposed a method that is based on local searches in the graphs to find pairs of connected modules. Ulitsky *et al.* (17) used a clustering of H as a starting point and then improved the solution by merging modules. An algorithm akin to (15) has been recently proposed for analyzing gene co-expression and DC networks. The joint analysis of these networks revealed gene groups that are much more (or much less) correlated in one class of individuals (24). Although previous algorithms for the module map problem proved valuable, a thorough analysis of the problem and of the merits and weaknesses of these algorithms in different scenarios is required.

The problem of finding an optimal module map is NP hard under most formulations, as it contains the clustering of H as a subproblem. Hence, heuristics are used. These algorithms usually contain two phases. We call the first phase ‘initiators’: algorithms for finding an initial solution that may contain many small modules. The second phase uses ‘improvers’: algorithms for improving an initial solution according to a predefined objective function. A variety of algorithms can be formed from different combinations of initiators and improvers.

Here, we study novel and extant initiators and improvers. We show that a new initiator based on maximal bicliques in G together with a statistically formulated global improver strategy performs consistently better or equal to extant methods on synthetic and real networks of several types. We call the resulting algorithm ModMap. We apply ModMap to experimental data in three biological scenarios: (i) using yeast PPIs and negative GIs, we find epistatic relations among protein complexes, (ii) using yeast PPIs and DNA damage-specific positive GIs, we detect emerging connections among protein complexes involved in DNA damage response and (iii) using DC analysis of gene expression profiles of non–small-cell lung cancer (NSCLC) tissues, we identify disease-specific loss of correlation between immune activation processes and detect disease-specific microRNAs.

## MATERIALS AND METHODS

### Definition of the module map problem

The input to the problem is a pair of networks H = (V,E_H_,W_H_) and G = (V,E_G_,W_G_) defined on the same set of vertices. These networks can be weighted or unweighted. The goal is to find a module map that summarizes both networks. A module map is a graph F = (M,L), where M is a collection of disjoint node sets, called modules, M = {M_1_, … , M_p_}, Mi 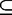 V, M_i _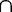 M_j_ = 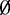, and L is a set of module pairs {(U_1_,V_1_), … , (U_p_,V_p_)}, where each U_i_ and V_i_ are in M. These pairs are called the map links. In addition, each module must be linked to at least one other module. Roughly speaking, our goal is to find a module map such that each module corresponds to a heavy subgraph of H, and each link represents a heavy bipartite subgraph in G between a pair of modules. A formal notion of heavy subgraphs will be introduced later. Figure 1D shows a toy example of two unweighted networks and their module map.

Previous algorithms for constructing module maps vary in the way they define the objective function and the links. The DICER algorithm (24) seeks one pair of linked modules at a time. A pair of modules is defined as linked if the sum of weights W_G_ between them is high enough. We call the approach of DICER ‘local’, as it finds one module pair at a time. The algorithm of Ulitsky *et al.* (17) aims to maximize the ‘global score’, namely, the total sum of scores within modules in H plus the sum of scores of links in G. In addition to increasing the global score, links between modules are accepted only if they pass a statistical significance test. We call the second approach ‘global’. Both methods identify the links and the modules simultaneously.

Figure 1D demonstrates the differences between the local and global approaches. Assume that in both graphs edge weights are 1, non-edge weights are −1 and that the local approach uses a threshold of 0 on the sum of W_G_ weights between two modules for reporting a link. In both approaches, modules are clusters of nodes with high density in H. According to both approaches, module 1 is linked to module 2: the local score is 4 (8 edges and 4 non-edges), the global analysis *P*-value for linkage is <0.05, and the total score for the module pair is 13 (module score 6 + 3 + link score 4). The sum of W_G_ weight between modules 2 and 3 is −4 (10 edges and 14 non-edges), and the local method rejects that link. However, the global approach will also link module 2 and 3: the linkage *P*-value is significant (*P* = 0.039), and adding this link will improve the global map score to 24 [13 for the (1,2) pair +15 for module 3–4 for the (2,3) link]. This example illustrates the advantage of the global approach on sparse graphs, in which large modules are not expected to be densely interconnected.

### Algorithms

We conducted a systematic study and developed further a family of two-phase algorithms for module map detection that find an initial solution (possibly consisting of many small modules) and then improve it. We call algorithms for the first phase initiators and algorithms for the second phase improvers. For simplicity, we describe the algorithms assuming that edges with positive weight are considered heavy. For unweighted graphs, we assume edge weights to be 1 and non-edge weights to be −1. For weighted graphs, all node pairs (edges) have weights, so there are no non-edges.

### Initiators

We tested five different initiators: (i) DICER (24), which finds one pair of linked modules at a time, (ii) hierarchical clustering of the graph H (25), which finds a set of modules, (iii) a greedy node addition algorithm for finding modules in H, (iv) DICER_k_ a variant of DICER wherein the minimum module size is set to k and (v) an algorithm based on enumeration of maximal bicliques in G using an exhaustive solver (26,27), followed by the cleaning process of DICER. We call the latter algorithm MBC-DICER, see Supplementary Text and Supplementary Figure S1 for a full description of all initiators. Each initiator creates an initial module set, but modules in the map constructed by clustering algorithms are not necessarily linked.

### Improvers

The ‘local improver’ (24) extends module map links by either adding a single node to a module or by merging two module map links. One drawback of this approach is that it cannot create new modules that are not represented in the initial solution. Another disadvantage is that it cannot merge a module whose two parts are linked to different modules that are unlinked. See Supplementary Figure S2 for examples. Later in the text we introduce the global improver, which can often overcome both problems.

Our ‘global improver’ is based on the procedure in (17). Let M = {M_1_, … , M_n_} be a collection of disjoint node sets (e.g. a set can be a single gene or not linked to any other set). Given sets (U,V), U, V 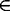 M and x 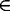 U, the significance of the linkage of x with V is calculated using Wilcoxon rank-sum test by comparing the edge weights W_G_ between x and V to the edge weights between x and all nodes not in V. Such *P*-values are calculated for all nodes in U and V, and they are combined using Stoufer’s method (28). If the final *P*-value p(U,V) is at most α then U and V are connected by a ‘link’ in the map. Let L = {(U_1_,V_1_), … , (U_p_,V_p_)} be the resulting set of links.

The ‘global score’ of the solution is the sum W_H_ of edge weights within each M_i_ plus the sum of W_G_ edge weights between the linked node sets:

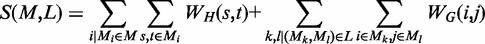


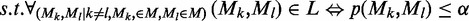



The improvement stage merges a pair of node sets (two modules or a module and a single gene) if the global score increases and the new link passes the significance test. Considering a merge that creates a new module Y requires recalculating p(Y,Z) for all other modules Z in M, in order to calculate the global score. This process is done greedily: iteratively, the merge that yields the best improvement is performed until no possible merge can improve the global score.

We modified the aforementioned method to allow for fast analysis of large graphs as follows. First, when calculating p(U,V), we consider the links in G’ (the unweighted version of G). We use a hypergeometric test to evaluate if a node has significant number of edges in G’ to the opposite set (e.g. from a node v 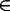 V to the set U), and then all node *P*-values are merged using Fisher’s method (29). The sets U and V are linked if the resulting value ≤α. This test is much faster and provides maps of equal quality to using the Wilcoxon test on G (see Supplementary Text). Weighted tests, such as the Wilcoxon test, are not always appropriate for detecting linkage among gene modules. For example, in the DC graphs, a strong link must contain many positive edges, whereas the Wilcoxon test only looks at the ranks of the edge scores.

Second, we set another parameter β>>α, and if at some point the *P*-value for the possible link between two sets is at least β, we say that the sets are ‘anti-linked’. In the original algorithm, when considering merging two sets U and V into W, possible links between W and every other set Y must be calculated. However, if U and Y are anti-linked or V and Y are anti-linked then we mark W and Y as anti-linked, avoiding the need to consider the possible link (W,Y). In practice, we used α = 0.005 for the yeast data as suggested in (17) and tested several options in the gene expression data (see Supplementary Text). In all cases we used β = 0.2. Finally, we perform multiple merge steps simultaneously in a single iteration in a way that guarantees that the global score improves (see Supplementary Text). This provides a speed up of two-fold or more in practice without loss of solution quality.

### Simulations

We constructed initially empty 500-node graphs H and G and then added edges creating a perfect module map in which modules are cliques in H and links are bicliques in G. The module map topology (M,L) was a random tree with |M| = 6. We then added two H-cliques and two G-bicliques to the graphs to represent additional ‘decoy’ structures that are not part of the map. Clique, biclique and module sizes were randomly selected in the range 10–20 with uniform distribution and disjoint node sets. Call the resulting edge sets E_H_* and E_G_*. Finally, we modified these graphs by introducing random noise: each edge in G and H was deleted with probability *P*, and each non-edge was replaced by an edge with probability *P*. All reversal steps were done independently. For creating weighted graphs, the same procedure was used, but all possible edges are present in the final H and G: w(u,v) is sampled from N(1,σ) if (u,v) is in E_H_* or E_G_*, and from N(−1,σ) otherwise. We also generated in this manner 1000 node graphs with 10 or 20 modules and five decoys (cliques and bicliques).

### Analysis of negative genetic interactions and protein-protein interactions in yeast

The PPIs and the negative GIs were downloaded from BIOGRID (30). These networks were used to find epistatic relations among protein complexes. The PPI network was used as H, and the GI network was used as G (see Supplementary Table S1).

### Analysis of DNA damage-specific genetic interactions data

We used the data of (21), in which all pairwise GIs among 418 genes were tested, and of (31), which tested GIs between 55 query genes and 2022 genes. A ‘DNA damage-specific positive GI’ was defined as one that had S < 0 in the untreated cells, S > 0.5 in the treated cells and the *P*-value for differential GI was <0.01. This analysis yielded 840 interactions from (21) and 1677 interactions from (31). We additionally defined a positive GI as ‘stable’ if it had S > 1.5 both in the untreated cells and in the DNA damage cells. This analysis provided 491 interactions in (21) and 3139 interactions in (31). Owing to the different experimental setups most of these GIs are not directly comparable.

### Calculating differential correlation scores

Given a training set containing gene expression profiles of subjects, we used the statistical method of (24) to compute for each gene pair its consistent correlation (CC) and DC scores. First, DC scores are computed using the real labels of the samples. Then, the scores are transformed to log-likelihood ratio (LLR) scores by comparing the original DC scores to scores calculated on the same data with randomly shuffled labels. Thus, positive LLR scores mark gene pairs with significant change in DC. The prior probability of real DC changes was set so that only correlation changes of at least 0.4 will have a positive LLR score. This approach guarantees a similar yet slightly more stringent acceptance threshold compared with (24). See Supplementary Text for additional information.

### GO and microRNA enrichment analysis

We used TANGO (32) for Gene Ontology molecular function and biological process enrichment analysis of modules and FAME (33) for microRNA enrichment analysis. Both tools are available as part of the EXPANDER software (34). When a set of modules was analyzed, we corrected for multiple testing using false discovery rate (FDR) with *q* = 0.05. The background set for the enrichment analysis was defined as the set of genes in the networks and not all genes in the organism. This filtering step reduces bias in case of overrepresentation of GO terms in the networks.

### Network visualization

Network visualization was done using Cytoscape (35).

### Availability

A command line tool for running ModMap is freely available for academic use at http://acgt.cs.tau.ac.il/modmap/.

## RESULTS

### Simulations

We first tested the different algorithms on synthetic graphs H and G. Starting from a perfect module map, we first added cliques in H and bicliques in G to represent additional structures that are not part of the map and then introduced random noise to the edges. To generate both sparse and denser graphs, we tested a wide range of the noise parameters σ and p in the weighted and the unweighted simulations, respectively (see ‘Materials and Methods’ section). The results presented here are for graphs with 500 nodes and six modules per map. We also tested larger graphs with similar results (see Supplementary Figures S3 and S4).

We tested 10 combinations of initiator and improver on 10 random data sets for each value of *P* and σ. We measured the quality of produced solutions using Jaccard coefficient between the reported modules and the known modules. The results of the unweighted and weighted models are shown in Figure 1A and B, respectively. Only the four algorithms that performed best on average in each simulation are shown. Supplementary Table S2 contains the results for all combinations. The local improvement algorithms did not reach perfect scores even on noiseless data. In contrast, MBC-DICER and DICER_5_ followed by global improver reached perfect Jaccard scores when there was no statistical noise. The high performance of MBC-DICER remained robust even when noise levels were as high as *P* = 0.15 in the unweighted model and σ = 1.2 in the weighted model. A comparison of all algorithms on unweighted graphs with 1000 nodes and 10 modules for noise level *P* = 0.15 is shown in Figure 1C. Performance remains high although the graphs are much larger. Using the improvers was beneficial compared with using only the initiator solutions, especially for the DICER variants. MBC-DICER with the global improver reached highest performance (0.87). Interestingly, the local improver was better than the global improver for all other algorithms (e.g. 0.71 versus 0.59 for DICER5). This is probably because the MBC-DICER initiator detects robust fully connected modules, which are a better starting point to the global improver at high noise levels. Tests with different values of k for the DICERk algorithm led us to choosing k = 5 (Supplementary Figure S4). In addition, we compared the performance of the global improver with the hypergeometric test and with the Wilcoxon rank-sum test, which was used in previous studies. Our results show that using the hypergeometric test reaches similar quality of results but is much faster (see Supplementary Text). Overall, the results indicate that MBC-DICER followed by the global improver achieved the best performance on both unweighted and weighted data. We call the resulting algorithm ModMap and will use it as the algorithm of choice from now on.

### Yeast protein-protein interaction and negative genetic interaction data

We used PPIs and negative GIs from BIOGRID (30) to find epistatic relations among protein complexes. Only genes that had both types of interactions were used. Overall, the networks contained 3979 genes, 45 456 PPIs, and 76 237 negative GIs (the interactions are listed in Supplementary Table S1). This number of genes and edges is larger than in previous studies. For example, (22) covered 1460 genes, and (17) covered 743 genes. Therefore, our networks have the potential to provide a broader overview of the yeast interactome and allow for a comprehensive performance testing of the different algorithms.

As done in previous studies, we evaluated solutions by their statistics and the functional characterization of the modules (17,22). The calculated solution statistics included the number of modules, the number of genes covered and the maximal module size. We used TANGO (34) to measure module functional enrichment, and reported the number of discovered GO terms, the percent of enriched modules and the percent of module map links for which both modules are enriched (with the same or with different functions), which we call ‘enriched links’. Enriched links represent dense GIs among known biological terms.

The solution statistics of all algorithms are shown in Supplementary Table S3. One can observe clear superiority of global over local improvers. In contrast to global improvers, which reported at least 100 modules and covered 800–1000 genes, the local improvers found 2–28 modules covering only 15–192 genes. Except for DICER, the results of all solutions were similar and of high quality. ModMap was the best in terms of the percent of enriched modules (87%) and percent of enriched links (80%). Taken together, the map of ModMap was best in combining functional comprehensiveness and quality. We also compared ModMap with other weighted approaches for GI data analysis (22,36) on the data of Collins *et al.* (37). See Supplementary Text for details. Our results show that ModMap produces high quality maps and improves on extant weighted approaches.

**Figure 2.**
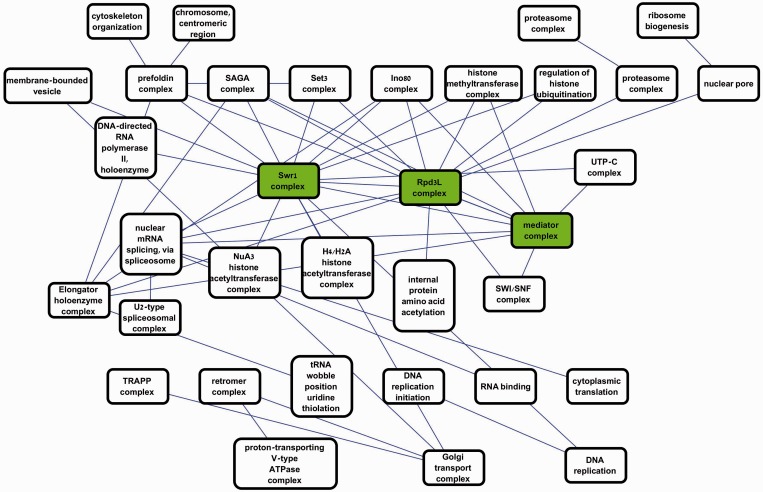
The yeast module map. Each node is a module in the yeast PPI network. The name of a node is the most significantly enriched GO term for that module. Each edge represents a highly significant link between two modules in the negative GI network (*P* < 1E-50). Modules that were not enriched for any GO term at 0.05 FDR are not shown. Three main chromatin-related hubs are marked in green. Some links connect disjoint modules enriched with similar GO terms (e.g. proteasome–proteasome link, top right), and other links show epistasis between different biological processes (e.g. nuclear pore and ribosome biogenesis, top right).

**Figure 3.**
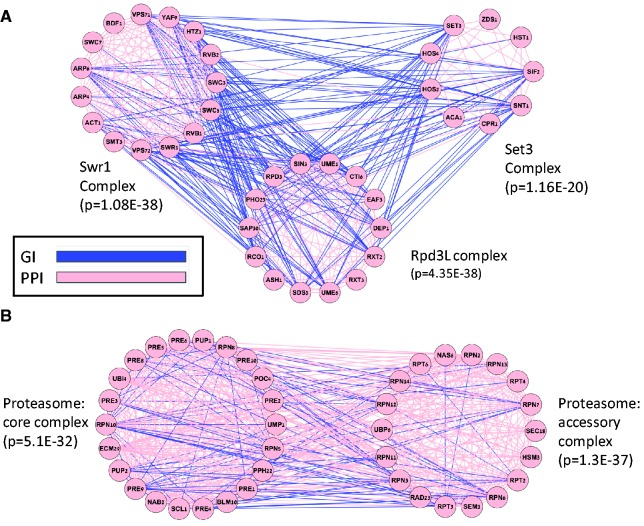
Examples of linked modules in the yeast module map. The genes of each module are arranged in a circle. Blue edges represent negative GIs and pink edges represent PPIs. For each module, the most enriched GO term is shown along with its enrichment *P*-value. (**A**) Linkage among different protein complexes. The significance of the links between Rpd3L and the Set3 complexes and between Swr1 and Rpd3L complexes is <10E-70. The link between Swr1 and Set3 is also highly significant (*P* = 4.29E-59). (**B**) Detection of subcomplexes. The joint analysis of the PPI and GI networks partitions the proteasome complex into its two subcomplexes: the accessory and the core complex.

Figure 2 shows a portion of the map constructed by ModMap where links were restricted to *P* < 10E-50 (for details see Supplementary Tables S4–S6). Each node represents a module, and edges represent map links. All modules in the presented map are enriched at 0.05 FDR with at least one GO term. The node labels show the most significantly enriched term. Three major hubs are marked in green: Rpd3L complex (14 genes, *P* = 4.35E-38), Swr1 complex (13 genes, *P* = 1.08E-35) and the mediator complex (17 genes, *P* = 4.89E-43). The Rpd3L and Swr1 complexes are chromatin related and were previously annotated as hubs of GIs in a gene-based study (38). Bandyopadhyay *et al.* (21) discovered some of the same links; however, module annotation there was manual, whereas our analysis was completely automatic and produced a much larger map. Moreover, our map extends on the previous observations by showing that the three hubs are linked and by providing additional links for the Rpd3L complex. In Figure 3, we focus on the three most significant links in the map (*P* < 1E-70). Figure 3A shows the connections between the Rpd3L and Set3 complexes and between the Rpd3L and Swr1 complexes. Rpd3L and Set3 are both histone deacetilases, and negative GI between them was reported in (20). The Rpd3L complex was split into two disjoint modules, whereas in our map it is detected as a single module, containing all 14 Rpd3L genes. Figure 3B shows a connection between two well-established subunits of the proteasome complex (39). This example shows how joint analysis of PPIs and GIs correctly detects core functional subunits even when they are connected by many PPIs.

### Analysis of DNA damage response networks in yeast

The module map described earlier in the text was obtained by analyzing the entire set of known negative GIs. Recent studies have gone beyond static analysis to detect changes in the GI network in response to DNA damage (21,31). In these studies, GIs were measured in untreated cells and following perturbation by the DNA-damaging agent methyl methanesulfonate (MMS) (40). We combined two such data sets (21,31) to detect ‘DNA damage-specific positive GIs’, i.e. differential positive GIs that emerge in the treated cells and are not observed in the untreated cells (see ‘Materials and Methods’ section). Negative GIs are typically observed between genes working in parallel, such as genes that are involved in two compensatory complexes or pathways that backup each other, and thus the loss of one is buffered by the other. Positive GIs are more likely to be observed between genes from the same complex or pathway, where most of the phenotypic effect is already observed in each single-knockout. Hence, DNA damage-specific positive GIs are expected to represent changes of the network in response to MMS, revealing DNA damage-specific interactions within pathways or between different pathways or complexes working in series. In total, 1078 genes were included in both studies, with 2227 DNA damage-specific positive GIs among them (see Supplementary Table S7). There were 6771 PPIs within that gene set.

We applied ModMap with the PPI network as H and the DNA damage-specific positive GI network as G. Because these networks were much smaller than in the previous analysis, we set the minimal module size to three. The small module sizes also affected the attainable *P*-values for links. Here, a pair of modules was defined as linked if its *P*-value was < 0.05 after Bonferonni correction, considering all statistical tests done by the algorithm during the improvement steps.

**Figure 4.**
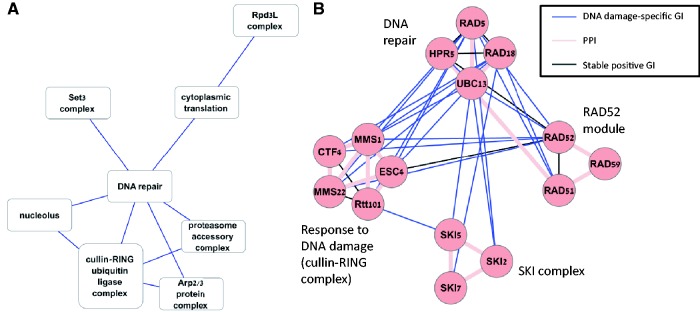
A module map of DNA damage-specific positive GIs. (**A**) A module map of the significantly enriched modules. Nodes represent modules and edges represent significant links (Bonferonni corrected *P* < 0.05). The name of a node is the most significantly enriched GO term. (**B**) A closer look at the DNA repair module and three-linked modules. Nodes represent genes and edges represent interactions: blue—DNA damage-specific positive GIs, pink—PPIs, black—stable positive GIs, which are observed both in the untreated and in the treated cells. This map shows the emerging connections between functional modules on DNA damage response covering DNA repair and checkpoint responses in the DNA repair module, response to damaged replication forks (the DNA damage response module), DNA double-stranded response genes (*RAD52* module) and RNA degradation-related genes (SKI complex module). The *RAD52* and SKI modules do not appear in A, as they reflect functions that do not have established GO terms.

The generated module map contained 78 genes in 12 modules, with 17 links among them. Module sizes ranged between 3 and 15. A complete description of the map is provided in Supplementary Tables S8–S10. A map of the modules that were significantly enriched with GO terms is shown in Figure 4A. The hub in this map is a module enriched with DNA repair genes, linked to six modules that cover a large variety of functions. In Figure 4B, we focus on the DNA repair-related module and on three of the modules linked to it. The DNA repair module contains four genes: *RAD5*, *RAD18*, *HPR5* and *UBC13*. Interestingly, although *UBC13* is known to physically interact with the three other genes, positive GIs that are consistently stable across experiments (see ‘Materials and Methods’ section) connect the other three genes, providing further evidence that the four genes are involved in a common process. The *RAD5*, *RAD18* and *UBC13* genes are known to be involved in post-replication repair (41–43) and *HPR5* is involved in checkpoint recovery (44,45).

The DNA repair hub module is linked to a module associated with response to DNA damage. It contains five genes: *CTF4*, *ESC4*, *MMS1*, *MMS22* and *Rt101*. The last four genes are part of the cullin-RING ubiquitin ligase complex (GO:0031461). The last three genes were shown to form a complex that stabilizes the replisome during replication stress (46,47). The *CTF4* gene is related to DNA repair and DNA replication initiation according to its GO annotations. The link suggests that this complex might work together with the DNA repair module for coping with damaged replication forks. Interestingly, the two MMS genes were originally detected in MMS sensitivity tests but are not expected to be required for double-stranded repair (47). The *RAD52* module (*RAD51*, *RAD52* and *RAD59*) is related to double-stranded DNA damage repair (48) and is linked both to the DNA damage repair module and to the DNA damage response module, suggesting these modules work together in the same pathway as a result of DNA damage to cope both with damaged replication forks and with double-stranded DNA breaks. The fourth linked module contains three genes of the SuperKiller (SKI) complex (*SKI2*, *SKI5* and *SKI7*). These genes are involved in 3–5 RNA degradation in the cytoplasmatic exosome (49,50). Our analysis suggests that this complex might also be involved in response to DNA damage. Previous studies have shown that RNA degradation cytoplasmatic genes might play a role in DNA damage response separately from their cytoplasmatic activity (51,52). The suggested roles of RNA degradation genes in DNA damage response include DNA stability and telomere stability related functionality (51), mediating the assembly of multiprotein complexes in double-stranded breaks (52) and specific mRNA degradation on DNA damage (53). Hence, our findings match prior studies and strengthen the role of the SKI complex in the response to DNA damage.

### Analysis of human co-expression and differential correlation networks

We applied ModMap on case-control gene expression data of NSCLC to reveal DC among highly correlated gene modules. The contribution of this part is two fold. First, we show that DC among gene modules is reproducible in cross-validation tests. Second, we analyze the map of DC patterns between gene modules discovered by ModMap.

Given a data set of gene expression profiles from cases and controls, we used the method of (24) to compute two scores for each gene pair: the CC score, which is positive if the gene pair is consistently correlated across phenotypes, and the DC score, which is positive if the correlation difference between the cases and controls is higher than expected by chance. These scores were then used as edge weights in networks H and G, respectively, on which a module map was learned. The methodology was evaluated using cross-validation: given a module map constructed on a set of profiles (the ‘training set’) and a disjoint set of samples (the ‘test set’), the quality of the predicted map was evaluated on the test set by comparing the DC of links and of non-links using Wilcoxon rank-sum test, where the null hypothesis is that there is no difference in DC between links and non-links. This measure is parameter-free and reflects all DC changes.

We tested several variants of the algorithm using 2-fold cross-validation. The maps produced by the local improver received low *P*-values but suffered from low coverage. For example, for the MBC-DICER initiator, the local improver achieved a *P*-value of 4.43E-4, but the map covered only 197 genes. In contrast, when applying ModMap (i.e. MBC-DICER with the global improver), the map covered 1289 genes, with *P*-value of 1.54E-10. Supplementary Text contains further results of testing different parameters of the global improver and tests on Alzheimer’s disease (54), which got similar cross-validation results. The full results are shown in Supplementary Table S11 for lung cancer and in Supplementary Table S12 for Alzehimer's disease. Taken together, ModMap produces large maps that are robust when tested on independent data sets.

**Figure 5.**
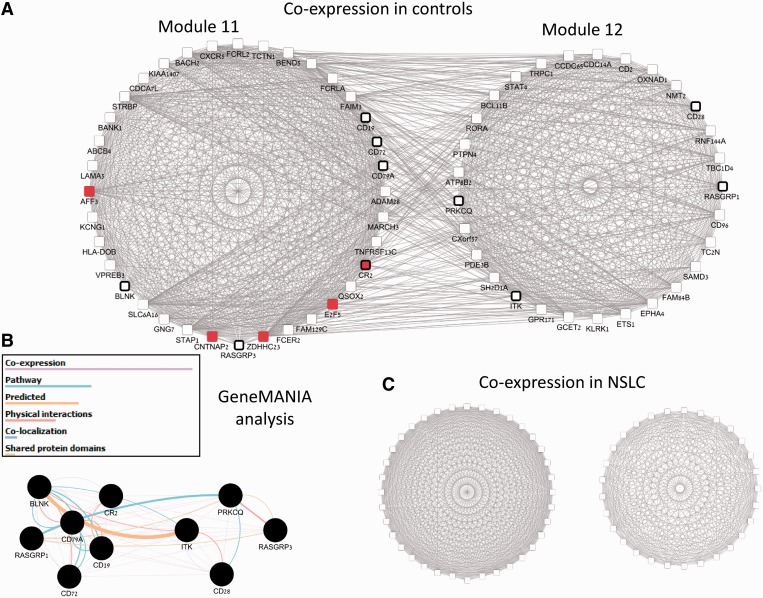
A pair of immune activation-related modules differentially correlated in NSCLC. (**A**) Two-linked modules, which are a part of the constructed module map. Nodes are genes and edges represent correlation >0.4 between the genes in the expression patterns of control class. Edges here correspond to high co-expression between two genes and do not reflect the weights in the CC or DC networks. We observe strong co-expression both within and between the modules. Nodes with black frames are related to immune activation response (six T-cell activation genes in module 11 and four B-cell activation genes in module 12). Red nodes in module 11 are targets of mir-34 family. (**B**) GeneMANIA analysis of the T-cell and B-cell signaling pathway genes shows that the genes of both modules are expected to interact in healthy controls. (**C**) The same two modules and their co-expression network in the NSCLC class. As in A, the genes within each module are highly co-expressed. In contrast to A, co-expression between the modules is completely diminished.

Next, we analyzed the module map obtained by running ModMap on all samples of the NSCLC data. The map covered 1921 genes in 76 modules, connected by 405 links (see Supplementary Tables S13 and S14 for details). To focus on strong changes in correlation between modules, we compared the DC of each link in the map to the DC calculated between random gene sets of the same sizes in 200 repeats and calculated the fold-change between the real link and the best random link as proposed in (24). The link fold-change scores are given in Supplementary Table S14. In all, 150 links had fold-change 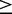
1.5, with the top five links exceeding 2.3. This indicates that the DC of the linked modules is far stronger than expected by chance. We also analyzed the modules of the top links using pathway enrichment analysis and microRNA enrichment analysis (see Supplementary Table S15 for details). One of the links connected two modules related to immune response activation. The linked modules are shown in Figure 5. In Figure 5A, we observe many high co-expression edges between the modules (gene pairs with r > 0.4) in the control class. Module 11 is enriched with B-cell receptor signaling pathway genes (6 genes, *P* = 3.1E-8). Module 12 is enriched with T-cell receptor signaling pathway genes (4 genes, *P* = 1.37E-4). Figure 5B shows GeneMANIA analysis of these 10 genes (7,55), which confirms that they are connected by several types of interactions. Figure 5C shows the co-expression of the same modules in the NSCLC class. Within each of the modules a strong level of co-expression is preserved, but the co-expression between the modules is abolished, suggesting that co-regulation of the different immune responses is lost in NSCLC. Finally, module 11 is highly enriched with targets of microRNA 34-a, b, c family (red nodes in Figure 5A), whose members are annotated as causal to NSCLC according to the mir-2-disease database (56). Taken together, these results show the ability of our analysis to detect NSCLC-related functional modules without using any prior knowledge.

## DISCUSSION

In this article, we presented a methodology for joint analysis of two gene networks, each representing a different type of omic relation between genes. The method identifies gene sets as modules and the complex structure of relations among them and summarizes the analysis in a module map. Modules correspond to interacting gene sets in the first network, and links in the module map correspond to interacting modules in the second. The map is constructed based on both networks simultaneously and thus can capture and reveal structures that are not identifiable when analyzing each data type separately. Our novel algorithms recovered the planted map structure in simulated data, even when the noise level in the data was high. We tested our methods in three biological applications: (i) yeast PPIs and negative GIs, (ii) yeast PPIs and DNA damage-specific positive GIs and (iii) DC analysis of human disease expression profiles. In all cases, certain parts of our maps are supported by prior biological knowledge, whereas other parts reveal novel structure and suggest new biological findings. The module map paradigm can be applied in principle on any two types of networks with underlying common nodes.

Our analysis of the yeast PPI and negative GI data constructed a large map describing epistatic relations among complexes. Our findings are in agreement with previous studies and show a complex map of interactions among chromatin modification-related complexes but also provide interactions with other functions, such as protein modification-related complexes. The analysis of the yeast PPIs and DNA damage-specific positive GIs produced a smaller map, which contains a DNA repair module as a central hub. The interactions of this module suggest that several mechanisms emerge simultaneously in response to MMS, including double strand repair, damaged replication fork repair and exosome complex activity. In the map constructed based on human NSCLC blood expression profiles, modules represent gene sets that are highly co-expressed both in cases and in healthy controls, whereas the map links correspond to specific rewiring of the co-expression network in NSCLC patients. In particular, we identified two modules enriched with immune activation genes manifesting a sharp drop in correlation in the NSCLC patients, suggesting diminished coordination between the T-cell and the B-cell enriched modules.

The concept of a module map can be viewed as a higher level combination of clustering and biclustering. Each of those problems has been extensively studied and was applied successfully to numerous single-type genomic and proteomic studies (1,57–68). By performing joint analysis on two different data types, we allow some relaxation of the objective function in each of the networks, for the sake of obtaining an overall clearer structure. Therefore, the new analysis can yield results when clustering or biclustering of one data type fails. One of the difficulties in clustering and biclustering is that module (or module-pair) sizes must be large enough to obtain highly significant sets. As our analysis demonstrates, the added power of the module map approach can identify relatively small precise groups that are beyond the detection ability of those prior methods.

Only a handful of studies have addressed the module map problem to date, and most of them focused on joint analysis of yeast PPI and GI networks. Ulitksy *et al.* (17) and Bandyopadhyay *et al.* (69) developed clustering methods that seek a map in which the likelihoods of the edge weights of PPIs and GIs within clusters or of GIs between linked clusters are higher than a given background distribution. Leiserson *et al.* (22,36) sought local maximum cuts in the weighted graph of the GIs by a greedy incremental approach, producing a collection of linked pairs of modules. Kelley and Ideker (20) developed a clustering algorithm that is based on graph compression, where the original GI graph is compressed to a module map. Hence, both (22,36) and (20) look for approximate bicliques that connect gene modules. In contrast, we enumerate the maximal bicliques of GIs, analyze them by taking into consideration the two interaction types to ensure that the initial solution contains dense strongly connected modules and improve the solution using our global improver. Because our approach is generic, it does not exploit the specific probabilistic nature of the GI data as other methods do (22,36). Nevertheless, we show that our method outperforms these and other extant methods in several criteria on GI data. In addition, because our algorithm is not limited by the type of the input data, we are able to combine many heterogeneous data sets (e.g. using all GIs of BioGRID) in our analysis.

When dissecting human expression profiles of disease patients and healthy controls, DC analysis was proposed as a way to discover gene modules whose inter-module correlation levels are altered in disease (12,14,24,70). We previously developed DICER (24), which uses a local approach to detect module pairs. Here, we go beyond it by finding maximal bicliques in the DC graph and by concurrently constructing a global map of modules. As we showed here, in most cases the map links are highly significant. However, we also observed cases where the absolute correlation change of modules might be mild even though the DC of the module pair is significant. A possible remedy is to give more emphasis to high absolute DC of map links so as to see the DC signal better. Another possible improvement is to enumerate bicliques using established heuristics [e.g. (68)].

A key factor in the performance of the ModMap algorithm is the objective function optimized. Here, we chose to maximize the sum of weights within modules plus the sum of weights of module links and assigned these weights based on a probabilistic model. On unweighted networks, such as the PPI and GI yeast networks, we set the weight of an edge to 1 and the weight of a non-edge to −1, thereby promoting strongly connected modules and links. This setting produced good results and revealed functional interactions among protein complexes. By setting different weights to non-edges in the graphs, future analyses can promote modules that are sparser, thus enabling better detection of interactions among complete pathways.

## SUPPLEMENTARY DATA

Supplementary Data are available at NAR Online.

## FUNDING

Israel Science Foundation [802/08 and 317/13]; Israel Cancer Research Fund; Lee Perlstein Kagan Charitable Trust (in parts). Azrieli Fellowship from Azrieli Foundation, Edmond J. Safra Center for Bioinformatics at Tel Aviv University, Israeli Center of Research Excellence (I-CORE), Gene Regulation in Complex Human Disease, Center No 41/11 (to D.A.); The funders had no role in study design, data collection and analysis, decision to publish or preparation of the manuscript. Funding for open access charge: Israel Science Foundation and I-CORE.

*Conflict of interest statement*. None declared.

## Supplementary Material

Supplementary Data
