# Supplementary material for: Constructing module maps for integrated analysis of heterogeneous biological networks
Source: Nucleic Acids Res. 2014 Jan 31;42(7):4208–19. doi: 10.1093/nar/gku102 (PMC3985673; doi:10.1093/nar/gku102)
Supplement: Supplementary Data [file supp_42_7_4208__index.html]

Constructing module maps for integrated analysis of heterogeneous biological networks — Constructing module maps for integrated analysis of heterogeneous biological networks — Supplementary Data 

# Constructing module maps for integrated analysis of heterogeneous biological networks

## Supplementary Data

files

**Files in this Data Supplement:**

- Supplementary Data - pdf file
- Supplementary Data - xlsx file
- Supplementary Data - docx file
