# Supplementary material for: Constructing module maps for integrated analysis of heterogeneous biological networks
Source: Nucleic Acids Res. 2014 Jan 31;42(7):4208–19. doi: 10.1093/nar/gku102 (PMC3985673; doi:10.1093/nar/gku102)
Supplement: Supplementary Data [file supp_gku102_nar-02861-n-2013-File007.pdf]

Supplementary Figure 1

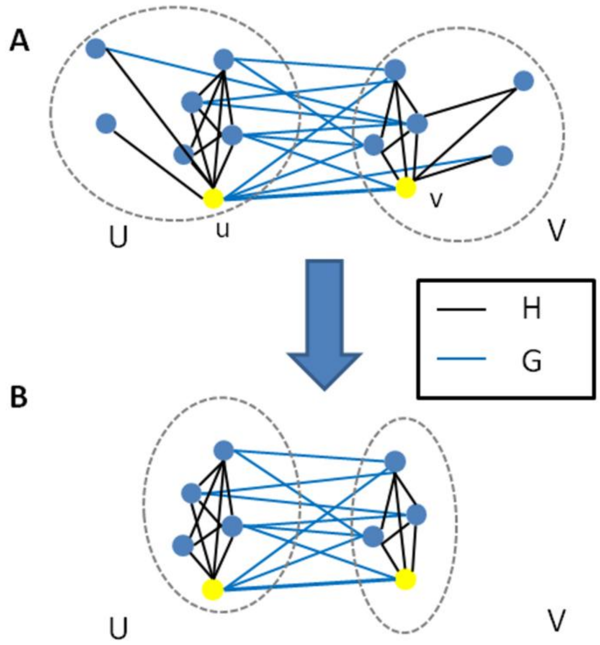

Supplementary Figure 1 Illustration of the DICER algorithm local search; A) An edge (u,v) in G is used as a starting point to form two sets U and V, which are the neighbors of u and v in H, respectively. B) Nodes are removed if they are not densely connected to their set in H or not densely connected to the other set in G. The final result after removing these nodes is shown – a pair of modules in H that are strongly linked in G.

Supplementary Figure 2

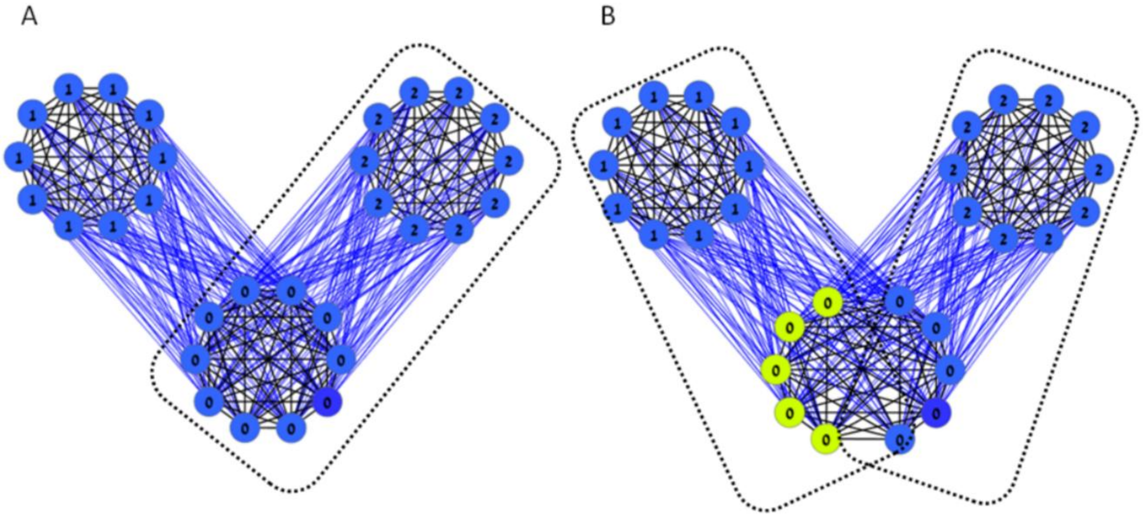

Supplementary Figure 2 Possible pitfalls of local improver that can be solved by the global improver. Edges of H are colored black; edges of G are colored blue. The number of a node is the module it belongs to. The initial solution that is provided to the improver is encircled by a dashed line. A) Given an initial solution that contains modules 0 and 2, a new module cannot be formed by the local improver. Hence, module 1 cannot be detected. B) Given an initial solution that partitions module 0 and links each part to a different module, the local improver cannot merge the two parts of module 0 since module 1 and module 2 are not linked.

Supplementary Figure 3

A) Un-weighted

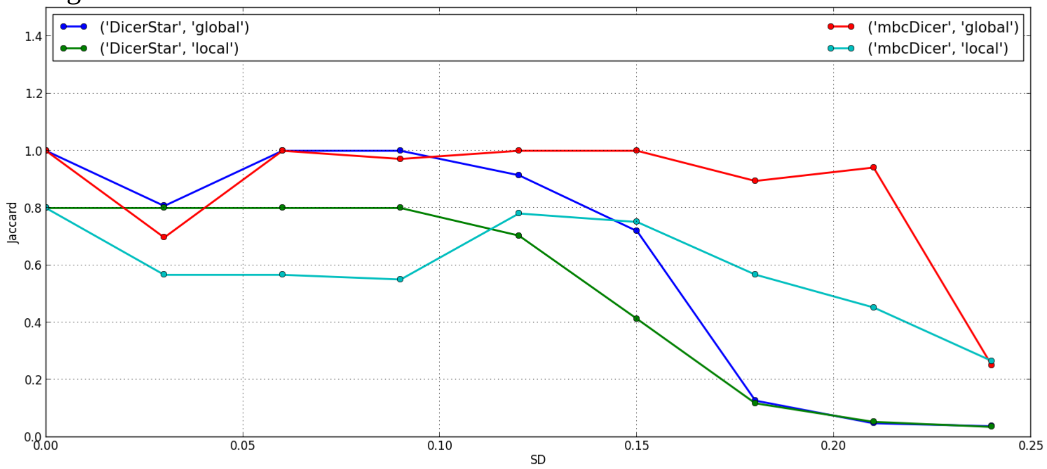

B) Weighted

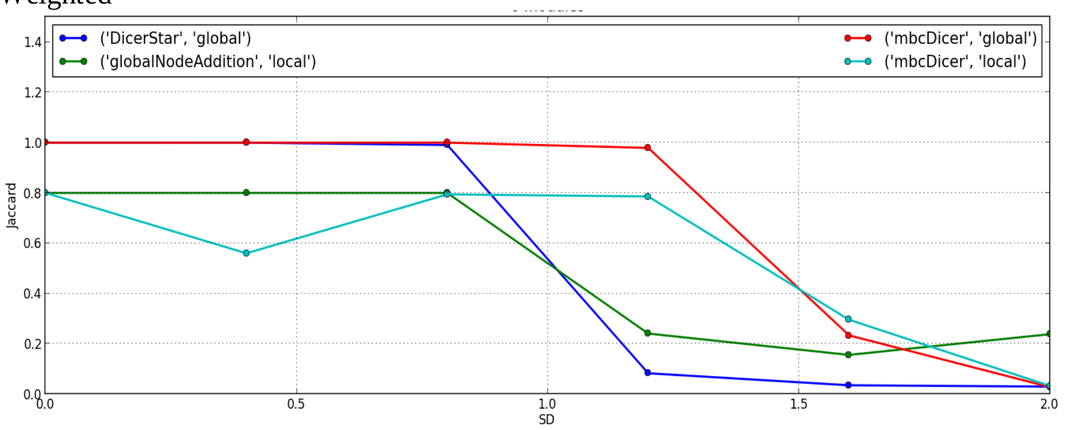

Supplementary Figure 3 Performance of module map algorithms on simulated data with 1000 nodes. A) Unweighted graphs. B) Weighted graphs. The 1000-node graphs contain an embedded module-map of six modules in a tree structure. In addition, random cliques and bicliques are embedded in the graphs. Module, clique, and biclique size is chosen uniformly at random between 10 and 20. In the un-weighted model each edge is replaced by a non-edge with probability p, and vice versa. In the weighted model edge weights are sampled from the normal distribution N(1,σ), and non-edge weights are sampled from the normal distribution N(-1, σ). A-B) The top four performing algorithms are presented. The y-axis shows the Jaccard coefficient between the output of the algorithms and the known modules.

Supplementary Figure 4

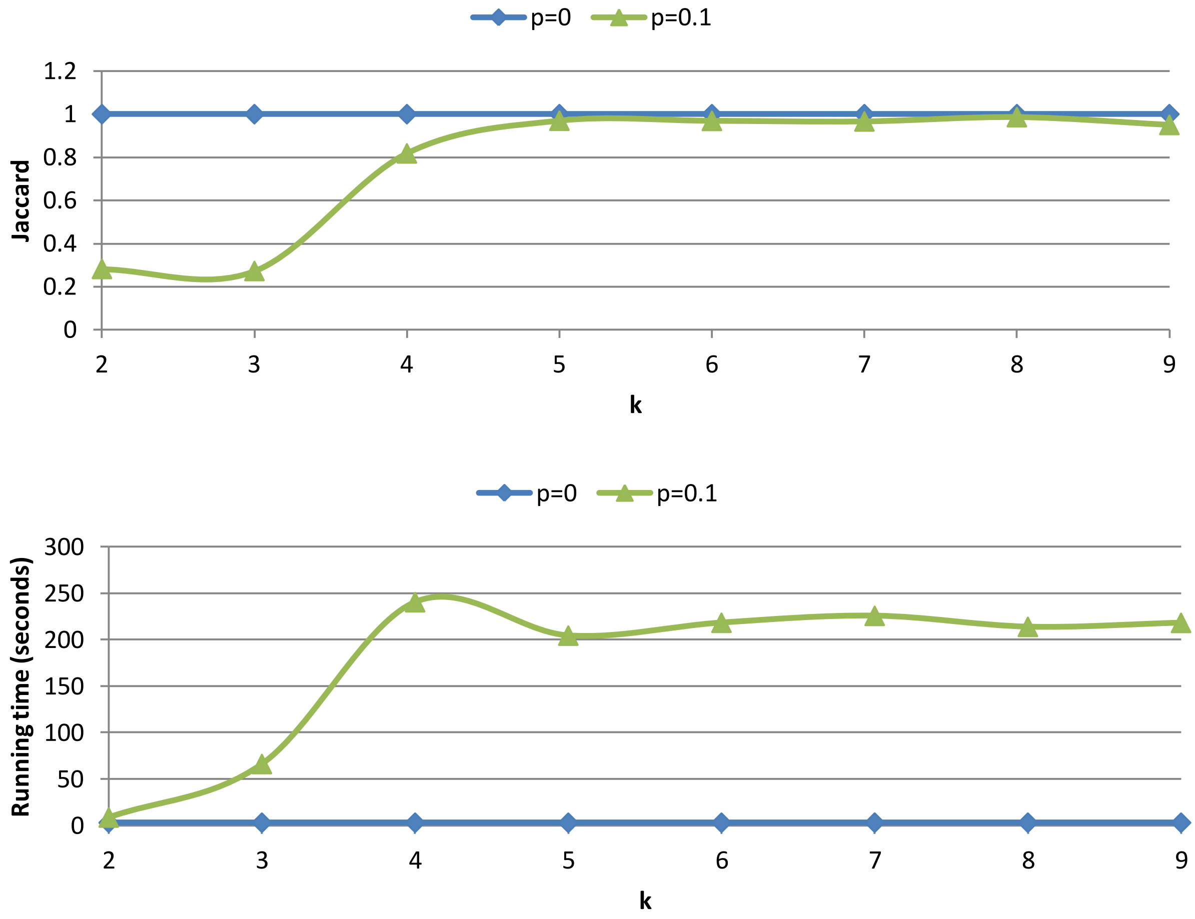

Supplementary Figure 4 Comparison of $DICER_k$ variants for different values of k on simulated unweighted data with 1000 nodes and 20 modules. A) Performance. B) Running times. The 1000-node graphs contain an embedded module-map of 20 modules in a tree structure. In addition, random cliques and bicliques are embedded in the graphs. Module, clique, and biclique size is chosen uniformly at random between 10 and 20. Each edge is replaced by a non-edge with probability p, and vice versa. The results show that using k=5 gives better performance than k<5, and that k>5 does not improve performance. Running times are very similar for k>3. Based on these results, since we expect biological data to contain both large and small modules, we concluded that using k=5 gives a good balance of quality and considering small modules, and used it in subsequent analyses.

Supplementary Text

Contents

Initiators....6

Proof of the guaranteed improvement during the iterations of the global improver 8

Comparison of scores for module links in the global improver 10

Comparison to other weighted approaches....11

Differential correlation cross-validation analysis 12

References 14

Initiators

We tested five different initiators. Three are based on previous methods and two are novel. The three extant initiators contained the DICER algorithm (23) and two clustering algorithms. We developed two additional initiators. The first is a modification of the DICER initiator, and is called DICER$_k$. The second utilizes an exhaustive solver for the maximal biclique problem (24,25) and is called MBC-DICER.

The DICER$_k$ initiator

The DICER initiator (23) starts from a positive edge (u,v) in G, and defines two node sets (U,V), where U is the set of (high weight) neighbors of u in H, and V is the set of neighbors of v in H. The goal is to remove nodes from U and V such that the resulting sets will constitute heavy sub-graphs of H and the weight of edges between U and V will be high in G. A simple example is shown in **Supplementary Figure 1**. Nodes that appear both in U and V are removed. In the next step, nodes in U and V are removed if this improves the score of the module map link in G or the module scores in H.

DICER works greedily, by iteratively removing a “bad” node, that is, a node that either has a negative sum of edge weights in H with its own group, or has a negative sum of weights in G with the other group. The total score of a node is the sum of the two scores. Nodes for which both G and H scores are negative are removed first, followed by other bad nodes, sorted by their total score. The process ends when there are no bad nodes. The resulting node sets U’ and V’ are accepted as modules only if each of them contains at least k nodes. In that case the nodes of U’ and V’ are removed from the graphs, and the process is repeated until no new module pair is found. In the original DICER algorithm we used k=2. Here we used k=5, which provided better results on real and simulated data (see Results).

The MBC-DICER initiator

We now describe an alternative method for constructing initial node sets U and V. Define an un-weighted graph G'= (V,E') with the same node set as G, and edge (u,v)∈E' if and only if W_G(u,v)>0. Two disjoint node sets (U,V) are called *fully connected* or a *biclique* in G' if every u ∈ U is connected to every v ∈ V. A biclique (U,V) is *maximal* if it is not a proper subset of another biclique. We search for maximal bicliques in G' using an exhaustive solver (24,25), restricting the search to maximal bicliques (U,V) such that |U|≥k and |V|≥k. Each such pair (U,V) is then subjected to the node removal procedure.

Since the number of maximal bicliques can be exponential (24,25) we use only the first 50,000 discovered bicliques as candidates for the node removal stage of the $DICER_k$ algorithm. Let S be a heap that contains the current set of candidate bicliques. We select the biclique (U,V) in S of maximal size |U|+|V| as the next candidate. The node removal stage produces from (U,V) a module pair (U',V'). If the latter is accepted, we remove the nodes of U' and V' from G' and from all bicliques in S, and remove bicliques whose new size is less than 2k. When S is empty we try to run the solver again. If the solver fails to find additional bicliques then the process is terminated.

Clustering algorithms

We included in our tests two clustering algorithms. Both look for clusters in H and disregard information from G. The first is complete-linkage hierarchical clustering (26). The second, which we call NodeAddition, starts with all nodes as modules, and repeatedly adds a singleton (a module with a single node) to a module if the sum of edge weights between them is the largest among all singleton-module pairs. This process is repeated until no singletons remain or until the best sum is negative.

Proof of the guaranteed improvement during the iterations of the global improver

Notations: The input to the problem is a pair of networks $H=(V,E,W_H)$ and $G=(V,F,W_G)$ defined on the same set of vertices. These networks can be weighted or un-weighted. The goal is to find a module-map that summarizes both networks. A *module-map* is a graph $F=(M,L)$ where M is a collection of disjoint node sets, called *modules*, $M=\{M_1,\ldots, M_p\}$, $M_i \subseteq V$, $M_i \cap M_j = \emptyset$, and L is a set of module pairs $\{(U_1,V_1), \ldots, (U_p,V_p)\}$, where each U_i and V_i are in M. These pairs are called the map *links* an express the set of significant links among modules according to some hypothesis testing function. In addition, each module must be linked to at least one other module.

The *global score* of the solution is the total sum W_H of edge weights within each M_i plus the total sum of W_G edge weights between each linked node set:

$$S(M,L) = \sum_i W_H(M_i) + \sum_{k,l \mid (M_k,M_l)\in L} W_G(M_k,M_l)$$

Where $W_H(M_i)$ is the total sum of weights within M_i in H, and $W_G(M_k, M_l)$ is the total sum of weights between M_k and M_l in G. The improvement stage merges a pair of node sets if the merge improves the global score. This process is done greedily: iteratively, the merge that yields the best improvement is performed until no possible merge can improve the global score.

We perform multiple merge steps simultaneously in a single iteration in a way that guarantees that the global score improves. Let L_i be the group of sets linked to M_i. Denote M_{ij} as the set resulting from merging M_i and M_j. Let L_{ij} be the group of sets linked to M_{ij} after performing the merge. Consider a case where two possible merges can improve the global score of a given solution (M,L): M_i with M_j, and M_a with M_b. If there is no overlap between the union of the sets M_i,M_j, L_i, L_j, L_{ij} and the union of the sets M_a,M_b, L_a, L_b, and L_{ab} then we say that $\{M_i,M_j\}$ and $\{M_a,M_b\}$ are *gain-independent.*

Theorem: When two possible merge steps $\{M_i,M_j\}$ and $\{M_a,M_b\}$ are gain-independent, performing both merge operations will improve the global score. Moreover, if the gain of the first merge is g_{ij} and the gain of the second merge is g_{ab} then the gain of performing both merges is at least $g_{ij} + g_{ab}$.

Proof: Let $M=\{M_1,\ldots, M_n\}$ be the partition of the node set before the merge, and let $L=\{(U_1,V_1), \ldots, (U_p,V_p)\}$ be the links, where each U_i and V_i are in M. The global score after merging M_i and M_j can be written as:

$$S_{new} = S(M,L) + W_H(M_i,M_j) - \sum_{M_k \in L_i} W_G(M_i,M_k) - \sum_{M_k \in L_j} W_G(M_j,M_k) + \sum_{M_k \in L_{ij}} W_G(M_{ij},M_k)$$

Thus, the gain can be written as:

$$g_{ij} = W_H(M_i,M_j) - \sum_{M_k \in L_i} W_G(M_i,M_k) - \sum_{M_k \in L_j} W_G(M_j,M_k) + \sum_{M_k \in L_{ij}} W_G(M_{ij},M_k) > 0$$

Note that under our assumptions of gain-independence this term does not involve any of the sets M_a,M_b, L_a, L_b, and L_{ab}. Therefore after merging M_a and M_b we get:

$$S_{new} = S(M,L) + g_{ij} + g_{ab} + \delta W_G(M_{ij},M_{ab}) \geq S(M,L) + g_{ij} + g_{ab}$$

Where δ=1 if M_{ij} is linked to M_{ab} and δ=0 otherwise. Thus, performing the additional merge between M_a and M_b would add g_{ab} to the new global score. The total gain is at least $g_{ij}+g_{ab}$ since we perform the merge steps without examining the possible link between M_{ij} and M_{ab}. ■

Corollary: A sequence of l merge steps can be performed simultaneously if the k'th merge in the sequence is gain-independent of merges 1 through k-1, for k=1,…,l. . ■

As a result of the theorem, instead of performing a single merge step and estimating the links on the new set we perform several merges, and evaluate the links between the new sets after merging. When we consider the merges in an iteration of the global improver, if many have a positive gain, we select the top B gains (we used B=1000). We then perform the set of merge steps ordered by their gain, skipping a merge if it is not gain-independent with all previous merges. We repeat this process until there is no merge that improves the global score.

While the asymptotic worst-case running time of this procedure is similar to performing a single merge at a time, we discovered that in practice many merge steps are performed per iteration. For example, in the lung cancer differential correlation analysis the maximal number of merges per iteration was 20, and the average was 4.

Comparison of scores for module links in the global improver

Our global improver used a statistical score to determine if two modules are linked. When the graphs are weighted, either the Wilcoxon rank-sum (WRS) test or the simpler hyper-geometric (HG) test can be used. We compared of the results of the global improver with each of the two scores using simulations. We generated weighted and unweighted graphs with 2000 nodes and 20 modules (see the main text for details). In each test, we ran the global improvers with both scores on the initial solution of DICER5. For graphs without any noise (i.e., the graph induces a perfect module map) the running times of the HG and WRS variants were 3 and 240 seconds respectively. Both variants perfectly discovered the planted module-map. On unweighted graphs, when the noise levels were increased to p=0.1, both algorithms reached the same performance of 0.97 but the HG running time was 3.8 seconds and the running time of the WRS variant was 394 seconds. We also applied the same test on weighted graphs with a standard deviation noise level of 0.8. The performance of the HG variant was 1 in 3.85 seconds. The performance of the WRS variant was 0.975 in 603 seconds. Our results show that the HG variant is much faster than the WRS variant, but achieves a similar performance.

Comparison to other weighted approaches

In our analysis in the main text we used un-weighted PPI and GI networks and included algorithms that are akin to previous methods (1-4). Other extant methods make use of the probabilistic scores of each GI edge, and incorporate both positive and negative GIs (5,6). Leiserson et al. (6,7) developed a method called Genecentric, which looks for locally maximum cuts in the GI graph. On the data of Collins et al (8), this method was reported to outperform other methods, including algorithms that integrate GI and PPI information (9,10). We compared the performance of our methods to Genecentric and the graph compression method of Kelley and Kingsford (5), on the Collins data. Note that the other methods use all GIs while our algorithm uses only the negative GIs of the Collins data. Genecentric solution contained 116 modules of average size 10.75. These modules were paired, so that the map contains 58 links. Kelley and Kingsford reported 117 modules of average size 3, and the map contained 403 links. The results are summarized in the table below. Kelley and Kingsford reported many small modules that are not significantly enriched after FDR correction. Thus, the percent of enriched modules and links is not high. The solution of Genecentric covered 1248 genes, whereas the ModMap solution covered only 238 (in 32 modules). The total number of enriched GO terms in our solution was 53, compared to 39 in Genecentric's solution. Finally, 79% of the links in our map were enriched, compared to only 43% in Genecentric. This comparison indicates that ModMap produces comparable or better maps than state of the art methods for analysis of GI data.

Comparison of ModMap to extant methods on the yeast PPI and GI data of Collins et al.

Algorithm	Number of modules	Gene coverage	Maximal module size	Number of enriched GO terms	Percent enriched modules	Number of links	Percent enriched links
**ModMap**	32	238	20	53	84	67	79
**Genecentric**	116	1248	25	39	63	58	43
**Kelley and Kingsford**	117	355	17	32	17	403	6

Differential correlation cross-validation analysis

Our tests on human data utilized expression profiles of lung cancer and Alzheimer's disease and matching controls in each dataset. The first tested dataset, GSE13255 (11), contained 256 peripheral blood mononuclear cells gene expression profiles of patients with non-small cell lung cancer (NSCLC, n=150) and controls (n=106). The second tested dataset, GSE15222 (12), contained 363 post mortem cortex gene expression profiles of Alzheimer's disease (AD) patients (n=176) and controls (n=187). Since the networks used in this analysis were completely different from these used in the yeast studies, we first re-evaluated the different algorithms on them, based on the ability to reveal major changes in co-expression between sick and healthy individuals.

We used the method of Amar et al. (4) to compute two log-likelihood ratio scores for each gene pair: the consistent correlation (CC) score is positive if the gene pair is consistently correlated across phenotypes, and the differential correlation (DC) score is positive if the correlation difference between the cases and controls is higher than expected by chance. These scores were then used as edge weights in networks H and G, respectively, on which a module map was learned.

Given a module map constructed on a set of profiles (the *training set*) and a disjoint set of samples (the *test set*), the quality of the map prediction was evaluated on the test set as follows. For each pair of modules we calculated the absolute average DC between the modules on the test set data, and compared the DC values for links and non-links (i.e., two modules in the map that are not linked) using the Wilcoxon rank-sum test, where the null hypothesis is that there is no difference in DC between links and non-links. This measure is parameter-free and reflects all DC changes. As an additional test, in order to focus on major DC changes, we ignored all links with DC < 0.4, removed unlinked modules and calculated the proportion and number of remaining modules, links and the gene coverage. These parameters reflect the overall predictive quality of each reported map, and its ability to find strong DC signals. We used 2-fold cross-validation, that is, half of the data served as the training set, and the other half served as the test set. The process was repeated with the roles of test and training set switched and results were averaged.

An important parameter in calculating the DC LLR scores is the prior probability of real DC changes. In (4) a parameter K controlled this prior probability. Given a value of K, the prior probability was set such that only DC scores that are distant from the mean of the random distribution by at least K standard deviations (of the random distribution) will get a positive LLR score. Informally, this process guarantees that if the difference between the real and random

distributions is minor, all LLR scores will be negative. In (4) a stringent approach was taken and the K parameter was set to 2. In this study we take a different, more direct approach to set the prior probability, using the following simple procedure: given a fixed threshold $\eta>0$ we set the prior to the maximal probability for which the LLR of η is negative. The intuition is that only DC of at least η receives a positive LLR score. Thus, unlike the K parameter, our approach is easily interpretable: we are guaranteed that absolute correlation changes lower than η will be assigned a non-positive LLR score. We used $\eta= 0.4$, which was equivalent to K $\cong$2.3 on the tested datasets. Thus, our criterion was even slightly more conservative than (4).

An important parameter of the global improver is α, which is used to determine if the link between two modules is significant. We tested several values for α: 1E-4, 1E-6, and 1E-8. For each combination of an initiator and a value of α, we evaluated the map using the Wilcoxon rank sum test as explained above. The performance of the different initiators as a function of α is shown in the table below. A clear advantage for α=1E-6 is observed. For this value, the p-values of all initiators except DICER remain significant after Bonferonni correction over all tests. In addition, a clear advantage for MBC-DICER (i.e., ModMap) is observed, achieving a p-value of 1.54E-10 in the lung cancer data, and 9.06E-6 in the AD data.

Algorithm	$-\log_{10}(\alpha)$	Lung cancer	AD
**DICER**	4	0.029494	9.02E-07
**DICER5**	4	0.003697	5.31E-07
**hierarchical**	4	0.23858	1.48E-07
**ModMap**	4	0.12515	4.69E-04
**NodeAddition**	4	0.471779	4.31E-04
**DICER**	6	1.38E-07	0.026652
**DICER5**	6	7.23E-07	4.21E-04
**hierarchical**	6	1.80E-10	1.57E-04
**ModMap**	6	1.54E-10	9.06E-06
**NodeAddition**	6	4.49E-05	0.00115
**DICER**	8	0.067356	0.019777
**DICER5**	8	0.021281	0.463172
**hierarchical**	8	0.373946	0.014721
**ModMap**	8	0.343799	0.110778
**NodeAddition**	8	0.394908	0.334608

The full cross-validation results for α=1E-6 are shown in **Supplementary Table 11** (NSCLC data) and **Supplementary Table 12** (AD data). The maps produced by the local improver received a very low p-value in the Wilcoxon rank-sum test between DC of map links and non-links, but suffered from low coverage. For example, for the MBC-DICER initiator, the local improver achieved a p-value of 4.43E-4 in the NSCLC data, and 3.31E-11 in the AD data. However, the map covered 197 genes in the NSCLC data, and 2197 genes in the AD data. In contrast, when applying ModMap (i.e., MBC-DICER with the global improver), the coverage was 1289 and 4955, respectively, with comparable p-values (1.54E-10, and 9.06E-6). Taken together, ModMap produces large maps that are robust when tested on independent datasets.

References

All references appear also in the main text.

1. Sharan, R., Ulitsky, I. and Shamir, R. (2007) Network-based prediction of protein function. *Molecular Systems Biology*, **3**.
2. Ulitsky, I., Shlomi, T., Kupiec, M. and Shamir, R. (2008) From E-MAPs to module maps: dissecting quantitative genetic interactions using physical interactions. *Molecular Systems Biology*, **4**.
3. Kelley, R. and Ideker, T. (2005) Systematic interpretation of genetic interactions using protein networks. *Nature biotechnology*, **23**, 561-566.
4. Amar, D., Safer, H. and Shamir, R. (2013) Dissection of Regulatory Networks that Are Altered in Disease via Differential Co-expression. *PLoS Comput Biol*, **9**, e1002955.
5. Kelley, D.R. and Kingsford, C. (2011) Extracting between-pathway models from E-MAP interactions using expected graph compression. *J Comput Biol*, **18**, 379-390.
6. Gallant, A., Leiserson, M.D., Kachalov, M., Cowen, L.J. and Hescott, B.J. (2013) Genecentric: a package to uncover graph-theoretic structure in high-throughput epistasis data. *BMC Bioinformatics*, **14**, 23.
7. Leiserson, M.D.M., Tatar, D., Cowen, L.J. and Hescott, B.J. (2011) Inferring Mechanisms of Compensation from E-MAP and SGA Data Using Local Search Algorithms for Max Cut. *J Comput Biol*, **18**, 1399-1409.
8. Collins, S.R., Miller, K.M., Maas, N.L., Roguev, A., Fillingham, J., Chu, C.S., Schuldiner, M., Gebbia, M., Recht, J., Shales, M. *et al.* (2007) Functional dissection of protein complexes involved in yeast chromosome biology using a genetic interaction map. *Nature*, **446**, 806-810.
9. Bandyopadhyay, S., Kelley, R., Krogan, N.J. and Ideker, T. (2008) Functional maps of protein complexes from quantitative genetic interaction data. *PLoS Comput Biol*, **4**, e1000065.

10. Ma, X.T., Tarone, A.M. and Li, W.Y. (2008) Mapping Genetically Compensatory Pathways from Synthetic Lethal Interactions in Yeast. *Plos One*, **3**.
11. Showe, M.K., Vachani, A., Kossenkov, A.V., Yousef, M., Nichols, C., Nikonova, E.V., Chang, C.L., Kucharczuk, J., Tran, B., Wakeam, E. *et al.* (2009) Gene Expression Profiles in Peripheral Blood Mononuclear Cells Can Distinguish Patients with Non-Small Cell Lung Cancer from Patients with Nonmalignant Lung Disease. *Cancer research*, **69**, 9202-9210.
12. Myers, A.J., Webster, J.A., Gibbs, J.R., Clarke, J., Ray, M., Zhang, W.X., Holmans, P., Rohrer, K., Zhao, A., Marlowe, L. *et al.* (2009) Genetic Control of Human Brain Transcript Expression in Alzheimer Disease. *American Journal of Human Genetics*, **84**, 445-458.

Supplementary Table 3: Performance of algorithms in the analysis of the yeast PPI and negative GI networks

Initial algorithm	improver	Number of modules	Mean module size	Gene coverage	Maximal module size	Num enriched terms	Percent enriched modules	Mean raw p-value	Percent enriched links	Num links in map
**DICER5**	global	103	9.29	957	46	249	0.82	3.4E-08	0.74	438
**DICER**	global	104	8.05	837	34	192	0.67	1.1E-07	0.61	498
**hierarchical**	global	123	7.13	877	30	186	0.68	1.6E-07	0.59	394
**ModMap**	(global)	100	9.46	946	49	243	0.87	4.7E-08	0.8	430
**Node Addition**	global	102	9.31	950	49	240	0.83	4.2E-08	0.79	430
**DICER**	local	6	8.17	49	12	15	1	8.7E-07	1	3
**DICER5**	local	28	6.86	192	9	54	0.82	1.3E-06	1	2
**hierarchical**	local	5	8	40	12	14	1	9.3E-07	1	3
**MBC-DICER**	local	4	8	32	9	10	1	2.4E-14	1	2
**Node Addition**	local	2	7.5	15	9	5	1	8.5E-14	1	1
**DICER**	-	606	2.75	1667	9	19	1	5.9E-07	-	-
**DICER5**	-	14	5.57	78	8	75	0.07	3.8E-07	-	-
**hierarchical**	-	1319	2.06	2722	10	200	0.07	1.4E-07	-	-
**MBC-DICER**	-	4	6	24	8	71	0.04	6.4E-07	-	-
**Node Addition**	-	1044	2.49	2600	49	7	1	6.5E-11	-	-

Supplementary Table 9: Enrichment analysis results on the ModMap modules in the PPI and DNA damage-specific GI data

Module	Category	p-value	Raw P-value	Gene IDs
**module_0**	DNA repair - GO:0006281	0.037	7.01E-05	[YJL092W, YDR092W, YCR066W, YLR032W]
**module_1**	proteasome accessory complex - GO:0022624	0.001	3.36E-17	[YOR117W, YKL145W, YFR052W, YDR394W, YGL048C, YPR108W, YBR272C, YDL007W]
**module_4**	Rpd3L complex - GO:0033698	0.001	1.79E-21	[YOL004W, YMR075W, YPL181W, YBR095C, YDR207C, YPL139C, YKL185W, YPR023C, YNL330C, YNL097C]
**module_5**	cytoplasmic translation - GO:0002181	0.001	6.80E-22	[YJL177W, YGL031C, YHR203C, YLR287C-A, YDL083C, YGL147C, YCR031C, YBR181C, YBL027W, YDR382W, YPL090C, YNL069C, YBL092W, YDR025W]
**module_6**	nucleolus - GO:0005730	0.001	1.05E-13	[YLR009W, YLR197W, YER002W, YDR496C, YOR272W, YFL002C, YDL014W, YPR016C, YMR049C, YOL077C, YNL061W]
**module_6**	preribosome - GO:0030684	0.001	2.41E-13	[YLR009W, YLR197W, YER002W, YOR272W, YFL002C, YDL014W, YPR016C, YMR049C, YOL077C, YNL061W]
**module_6**	ribosome biogenesis - GO:0042254	0.001	4.50E-13	[YLR009W, YLR197W, YER002W, YDR496C, YOR272W, YFL002C, YDL014W, YPR016C, YMR049C, YOL077C, YNL061W]
**module_6**	ribosomal large subunit biogenesis - GO:0042273	0.001	3.86E-12	[YLR009W, YER002W, YDR496C, YOR272W, YFL002C, YPR016C, YMR049C, YOL077C]
**module_6**	preribosome, large subunit precursor - GO:0030687	0.001	7.40E-11	[YLR009W, YER002W, YOR272W, YPR016C, YMR049C, YOL077C, YNL061W]
**module_7**	cullin-RING ubiquitin ligase complex - GO:0031461	0.001	2.70E-07	[YLR320W, YJL047C, YHR154W, YPR164W]
**module_7**	DNA-dependent DNA replication - GO:0006261	0.005	5.72E-06	[YPR135W, YLR320W, YJL047C, YPR164W]
**module_7**	response to DNA damage stimulus - GO:0006974	0.016	1.72E-05	[YPR135W, YLR320W, YJL047C, YHR154W, YPR164W]
**module_8**	Arp2/3 protein complex - GO:0005885	0.001	1.34E-09	[YDL029W, YNR035C, YJR065C, YIL062C]
**module_8**	actin cortical patch - GO:0030479	0.001	1.69E-09	[YDL029W, YNR035C, YJR065C, YIL062C, YLR337C]
**module_9**	DNA replication-independent nucleosome assembly - GO:0006336	0.001	4.99E-13	[YJR140C, YBR215W, YBL008W, YJL115W, YNL206C]
**module_11**	Set3 complex - GO:0034967	0.001	1.34E-09	[YBR103W, YCR033W, YGL194C, YKR029C]
**module_11**	covalent chromatin modification - GO:0016569	0.001	1.42E-07	[YBR103W, YCR033W, YHR119W, YGL194C, YKR029C]

Supplementary Table 10: The module links of the ModMap solution in the PPI and DNA damage-specific GI data

Module A	Module B
module_1	module_0
module_2	module_0
module_3	module_2
module_5	module_0
module_5	module_4
module_6	module_0
module_6	module_3
module_7	module_0
module_7	module_1
module_7	module_2
module_7	module_6
module_8	module_0
module_8	module_7
module_9	module_2
module_9	module_3
module_10	module_0
module_11	module_0

Supplementary Table 11: Cross-validation results in the NSCLC data

		Original solution						Ignoring DC below 0.4 in the test set			
Initiator	Improver	Gene coverage	Mean module size	Median module size	Number of map links	Number of modules	Max module size	Gene coverage	Number of modules	Number of map links	Pvalue
DICER	global	2446.5	15.75	8	1022.5	156	196.5	1578	90	9.10E+01	**1.38E-07**
DICER5	global	2101	19.24	9	853	111	200.5	1464.5	70.5	93	**7.23E-07**
hierarchical	global	2642	18.50	9.5	892	142.5	252	1677.5	77.5	90	**1.80E-10**
ModMap	(global)	1939.5	21.38	9	622	92	220.5	1289.5	57	67	**1.54E-10**
Node Addition	global	2617	20.44	8	788	128.5	260	1488.5	69.5	77.5	**4.49E-05**
DICER	local	1613	8.87	7	102	181.5	52.5	238.5	22.5	2	**3.30E-07**
DICER5	local	1105	12.51	8.5	113	88	55.5	261.5	25	19	**6.79E-06**
hierarchical	local	535	14.87	11	32	36	59.5	160.5	11	8	0.013
MBC-DICER	local	724	15.03	10.5	70.5	50	46.5	197	15.5	11	**4.43E-04**
Node Addition	local	331	15.52	16	24.5	22	45	29	3	2	0.065

Supplementary Table 12: Cross-validation results in the AD data

		Original solution						Ignoring DC below 0.4 in the test set			
Initiator	Improver	Gene coverage	Mean module size	Median module size	Number of map links	Number of modules	Max module size	Gene coverage	Number of modules	Number of map links	Pvalue
DICER	global	5219.5	62.26	16.5	787.5	84	534	4324	65	112	2.67E-02
DICER5	global	5214.5	64.03	14.5	783.5	82	544	4642.5	65	110	**4.21E-04**
hierarchical	global	5703	55.48	14	1188.5	103.5	747.5	4770.5	74	142	**1.57E-04**
ModMap	(global)	5360	70.55	22	908	76	649.5	4955.5	62.5	122.5	**9.06E-06**
Node Addition	global	5610	64.57	10.5	781.5	87	1052	4969.5	60	112	**1.15E-03**
DICER	local	4057.5	25.16	11	148.5	161.5	302.5	1934	52.5	55	**0**
DICER5	local	3980.5	30.00	12.5	216.5	134	318	2531	70.5	75.5	**8.88E-16**
hierarchical	local	3709.5	34.82	16.5	225.5	106.5	250	2155	51	55.5	**7.69E-12**
MBC-DICER	local	3906.5	34.58	18	249.5	114	218	2197	60.5	62	**3.31E-11**
Node Addition	local	1793.5	91.63	50	23	19.5	328	1161.5	11.5	7.5	7.30E-02

Supplementary Table 15: Enrichment analysis of the modules in the ModMap solution in the NSCLC data

Module	Term	# genes	Raw p-value	Corrected P-value	Genes
			**KEGG**		
module_11	Primary immunodeficiency	4	8.13E-06	3.58E-04	[CD19, TNFRSF13C, CD79A, BLNK]
module_11	B cell receptor signaling pathway	6	3.03E-08	2.54E-06	[CR2, CD19, RASGRP3, CD79A, CD72, BLNK]
module_12	T cell receptor signaling pathway	4	1.37E-04	0.0161	[ITK, PRKCQ, RASGRP1, CD28]
module_20	Glycolysis / Gluconeogenesis	4	1.60E-05	0.00113	[HK3, ALDH2, FBP1, ALDH3B1]
module_20	Pentose phosphate pathway	2	0.00275	0.0961	[PGD, FBP1]
module_3	mTOR signaling pathway	2	6.43E-04	0.0392	[HIF1A, PIK3CB]
			miRNA		
module_11	mir-34a/34b-5p/34c/34c-5p/449/449abc/699	5	8.45E-04	0.002	[ZDHHC23, CR2, E2F5, AFF3, CNTNAP2]
module_11	mir-33/33ab	3	0.0114	0.0075	[BACH2, AFF3, STRBP]
module_2	mir-125/351	9	0.053	0.0015	[PACS2, ST6GALNAC6, RABEP2, ARHGEF1, MED15, ULK3, MKNK2, LYPLA2, KLC2]
module_2	mir-204/211	7	0.148	0.002	[GET4, AP2A2, BCL9L, BIN1, SDHAF1, DNM2, RIN3]
module_3	mir-340/340-5p	3	0.048	0.011	[CDC42SE2, JAK1, HERC3]
module_33	mir-124/506	3	0.0278	0.046	[NUMA1, ABHD4, BMF]
