# Supplementary material for: Constructing module maps for integrated analysis of heterogeneous biological networks
Source: Nucleic Acids Res. 2014 Jan 31;42(7):4208–19. doi: 10.1093/nar/gku102 (PMC3985673; doi:10.1093/nar/gku102)
Supplement: Supplementary Data [file supp_gku102_nar-02861-n-2013-File009.docx]

**Supplementary Informatio**n

**Supplementary Figure 1:** Illustration of the DICER algorithm local search

**Supplementary Figure 2:** Possible pitfalls of local improver that can be solved by the global improver

**Supplementary Figure 3:** Performance of module map algorithms on simulated data with 1000 nodes and 20 modules.

**Supplementary Figure 4:** Performance of DICER_k_ variants on simulated unweighted data with 1000 nodes and 20 modules

**Supplementary Table 1:** Yeast interactions used in the analysis of PPI and negative GI networks

**Supplementary Table 2:** Results of the simulations

**Supplementary Table 3:** Performance of algorithms in the analysis of the yeast PPI and negative GI networks

**Supplementary Table 4:** ModMap solution on the yeast PPI and negative GI networks

**Supplementary Table 5:** Enrichment analysis of the ModMap solution on the yeast PPI and negative GIs networks

**Supplementary Table 6:** The highly significant module links in the ModMap solution on yeast PPIs and negative GIs networks

**Supplementary Table 7:** The set of positive GIs that are specific to yeast cells treated with DNA damage agent MMS

**Supplementary Table 8:** ModMap solution on the PPI and DNA damage-specific GI data

**Supplementary Table 9:** Enrichment analysis of the ModMap modules in the PPI and DNA damage-specific GI data

**Supplementary Table 10:** The module links of the ModMap solution in the PPI and DNA damage-specific GI data

**Supplementary Table 11:** Cross-validation results in the NSCLC data

**Supplementary Table 12:** Cross-validation results in the AD data

**Supplementary Text**

**Supplementary Table 13:** ModMap solution on the NSCLC data

**Supplementary Table 14:** Module map links of the ModMap solution in the NSCLC data

**Supplementary Table 15:** Enrichment analysis of the modules in the ModMap solution in the NSCLC data
